# Description and Utilization of the United States Department of Defense Serum Repository: A Review of Published Studies, 1985-2012

**DOI:** 10.1371/journal.pone.0114857

**Published:** 2015-02-27

**Authors:** Christopher L. Perdue, Angelia A. Eick Cost, Mark V. Rubertone, Luther E. Lindler, Sharon L. Ludwig

**Affiliations:** Armed Forces Health Surveillance Center, Silver Spring, Maryland, United States of America; Fudan University, CHINA

## Abstract

Specimens in the United States Department of Defense (DoD) Serum Repository have accumulated in frozen storage since 1985 when the DoD began universal screening for human immunodeficiency virus. Use of the stored serum for health research has been carefully controlled, but the resulting publications have never been systematically identified or described. The Armed Forces Health Surveillance Center (AFHSC) information systems and open (online) sites were used as data sources. Through 2012, the repository contained 54,542,658 serum specimens, of which 228,610 (0.42%) have been accessed for any purpose. Between 2001 (the first year that comprehensive, digital records were available) and 2012, 65.2% of all approved requests for serum were for healthcare or public health investigations, but greater than 99% of all shipped samples were for research. Using two different methods – a structure search of PubMed and an exhaustive online search based on records from AFHSC – we identified 76 articles published between October 1988 and March 2013 that covered a multitude of infectious diseases, injuries, environmental exposures and mental health conditions through analysis of antibodies, biological metabolic, signaling and regulatory substances, Vitamin D, organochlorines, dioxin, omega-3-fatty acid, and portions of human deoxyribonucleic acid. Despite its operational and scientific value, it appears that the DoD Serum Repository has been underutilized. Changes to policy and increased capacity for specimen processing could increase use of the repository without risking privacy or the availability of specimens for the healthcare of individual service members in the future.

## Background

As early as the 1950s, the Department of Defense (DoD) collected serum from military service members to conduct militarily relevant epidemiologic studies, inform health policy, and enhance the health and operational strength of the force, however these specimens were not reposed in a central repository nor made available for general medical or operational research.[1,2,3,4,5] The DoD began long-term holdings of frozen human serum specimens in 1985 after the start of universal, mandatory screening for human immunodeficiency virus (HIV) among applicants to military service prior to induction; and among service members periodically throughout their time in service.[6] Initially, the DoD serum holdings were maintained separately by the Navy (combined with the Marine Corps),[7] the Army,[8,9] and the Air Force[10].

In 1989, the Army and the Navy/Marine Corps repositories were physically combined at the Walter Reed Army Institute of Research. A comprehensive, systematic inventory system that covered the entire collection was implemented in 1994 after the responsibility for the combined repository was transferred to the Army Medical Surveillance Activity (AMSA).[6] At that time, records for individuals who provided serum were also associated with information in the Defense Medical Surveillance System (DMSS), an epidemiologic database containing military service records, deployment histories, demographic data, administrative healthcare data from the military health system (including paid civilian healthcare), and immunization records. Serum samples from the Air Force were added in 1996 marking the official beginning of the DoD Serum Repository (DoDSR). The DMSS and DoDSR, along with the responsibility to conduct DoD-wide health surveillance, were transferred to the Armed Forces Health Surveillance Center (AFHSC) in 2008.[11] While responsibility for maintaining the DoD serum bank belongs to AFHSC, the individual Services are responsible for maintaining the facilities and personnel required to draw, process and test specimens as needed. AFHSC takes responsibility for the specimens only after sera are separated from the rest of the blood content and frozen at one of the central specimen processing laboratories.

To our knowledge, the DoDSR is the largest bank of human serum in the world. It is maintained at a constant temperature of -30°Celsius (C) and has redundant cooling and electrical systems. [6] Since the DoDSR’s inception, access to specimens has been controlled by restricting physical access to the repository and careful review of all requests for serum. DoD Directive 5400.11, September 2011, “DoD Health Information Privacy Regulation” and DoD Instruction 6025.18, December 2009, “Privacy of Individually Identifiable Health Information in DoD Health Care Programs” (among others) require military health system employees and facilities, including the DoDSR, to comply with US privacy laws. All requests for DoDSR specimens are reviewed and approved (or rejected) by an internal committee of scientists at AFHSC. There is a distinction made between studies based on operational requirements—those intended to respond to a public health problem or for individual healthcare—and research studies intended to result in generalizable knowledge.

The review process verifies the appropriateness of the request and—for public health investigations and research—determines that serum samples and supporting epidemiologic data are available to satisfy the study design. Access to specimens for operational requirements is limited to treating clinicians (military or civilian) or military scientists conducting investigations with command approval. Clinical requests for serum must include a signed form indicating the patient’s consent or proxy consent by the attending physician. Use of the DoDSR for research is restricted to studies with at least one military principal investigator and a protocol that has been approved by a military human subjects review board (HSRB).[12] AFHSC does not maintain its own HSRB.

The DoD laboratories begain keeping the serum that remained following HIV testing primarily to support other HIV-related studies. In 1988, Burke and colleagues published the first (known) retrospective serological study using the frozen serum holdings in which they evaluated the false positive rate of HIV screening procedures of the time.[13] Roberts and his colleagues published the first (known) non-HIV-related study in 1992 in which they established the rates of human T-lymphotropic virus I and II infections among those applying for military service.[14] Other early retrospective serum studies were used to inform military vaccination policies for measles and rubella,[15] Japanese encephalitis virus,[16] adenovirus (serotypes 4 and 7),[17] and tick-borne encephalitis virus.[18]

In recognition of the DoDSR as an extraordinary resource for force health protection, and in response to controversies related to a potential Gulf War syndrome,[19,20,21] the Deputy Secretary of Defense for Health Affairs issued DoD Directive 6490.02 in 1997, encouraging use of the DoDSR for “medical surveillance for clinical diagnosis and epidemiologic studies… for the identification, prevention, and control of diseases associated with operational deployments of military personnel” (quoted from page 4). A companion instructional document established the requirement for additional serum collection (beyond the HIV screening requirements) from each service member immediately before and after deployments, increasing the ability of military scientists to focus on deployment-related exposures and health outcomes. In 2004, DoD Directive 6490.02 was re-issued but with modifications to promote studies related to any aspect of military service (not just deployments). That directive was re-issued again in 2007 and 2012 as DoD Directive 6490.02E “Comprehensive Health Surveillance.”[22]

The ages of service members at the times of specimen collection and the timing between specimens are also important factors for study design, though such details have not been previously documented in open literature. Regarding past uses of the DoDSR, it is relatively easy to identify published studies that used specimens from the DoDSR, but there has been no systematic attempt to identify all such studies that were published. Through a systematic process, taking advantage of digital records maintained at AFHSC, we attempted to find all of the published articles for studies that used specimens from the DoDSR (or its predecessors). In reviewing those articles, we sought to understand the breadth and depth to which specific topics, diseases or conditions had been studied; and draw conclusions about the use of the DoDSR to date. We also used the available databases to describe the age distribution of specimens in the DoDSR.

## Methods

AFHSC databases were queried to determine the number of serum specimens placed into storage each year as well as the ages of service members at the time of specimen collection. At no time during this project did we seek to obtain personal health data or individually identifiable information. Serum specimens were not accessed.

Algorithmic search method: we initially attempted to identify DoDSR-related publications using a relatively restricted search of the *PubMed* online database (National Center for Biotechnological Information, http://www.pubmed.org). The text strings “DoDSR,” “DoD serum repository,” “military (or defense) stored (or frozen or banked) serum,” “military (or defense) seroepidemiology,” “military (or defense) seroprevalence,” and “military (or defense) serosurvey” (in English) resulted in 383 unique citations. Forty-eight (48) met the screening criteria—a study conducted by or for the US military using a retrospective serological study design—and were read in full. Reading two of the articles was insufficient to determine that the research used serum from the repository, so the primary authors were contacted; neither of those studies used DoDSR specimens. From our first search method, we identified 25 publications to include in this review.

Intense search method: to ensure that we found as many publications as possible, we obtained digital records of studies from AFHSC’s databases that were supported between 1996 and 2012. For earlier years, only archived paper and email records now exist, and those could not be systematically searched at the time of our review. It should be noted that the current DoDSR inventory management database contains records for all of the specimens collected since 1985. According to the available records, there were 140 serum requests that had been granted (i.e. specimens were removed from the repository and sent to the investigator) between 1996 and 2012. For each request, we used the name of the investigator (or co-investigators if any were available), the date that serum specimens were retrieved and the health condition(s) that the investigators said they were researching to search PubMed (again), the DoD’s online Defense Technical Information Center (DTIC; http://www.dtic.mil) and Google (http://www.google.com). We searched those sources exhaustively and ultimately discovered another 51 articles that used DoDSR specimens. We did not systematically contact investigators who had received serum specimens to determine whether their study had been (or was expected to be) published. This was due to the high mobility of service members and difficulty in contacting individuals years later and because there was no requirement for investigators to notify the AFHSC of publications until 2013. We stopped searching for publications in April 2013 because of limitations in available resource, and included only those that were published through March 2013. There were three studies identified using the algorithmic search method that were not found using the intense search method because AFHSC records prior to October 1996 (the date that the current database was started) were not available.

Seventy-six publications were reviewed to determine the analyte(s) and health condition(s) that were the subject of investigation. No attempt was made at meta-analysis or other statistical observations. We included publications in this review without *a priori* or *post hoc* judgments regarding study design, strength of the conclusions, citation indices or journal source. A number of poster presentations and conference abstracts (independent of a related publication) were discovered, but those were not included primarily because full details about the study design, methods and results would not necessarily be available to the average scientific reader. This review was limited to studies that required serum to be taken from the DoDSR after freezing and did not include any publications that resulted from laboratory testing before banking. We did not consider the method by which articles were discovered (by us) as an important factor in our attempt to understand the historical (and potential future) utility of the DoDSR.

## Results

### Contents of the DoDSR

As of the end of 2012, there were 54,542,658 specimens in the DoDSR, of which 228,610 original vials (0.42%) had been accessed for either a research or operational purpose. Since 1989 when HIV screening programs were fully implemented, the DoD’s frozen serum holdings grew by a mean of 2.12 million specimens annually (standard deviation [SD] = 202,995). From year to year, there were the expected increases and decreases in the number of specimens entering the repository related to the number of military deployments (data not shown). The repository experienced the greatest annual accumulation in 2003 of over 2.53 million specimens associated with the beginning of Operation Iraqi Freedom.

Dropping outliers who, according to AFHSC records, were apparently younger than 17 years or older than 70 at the time that serum was collected (e.g. not actually eligible for military service), the mean age of service members at the time of specimen collection between 1985 and 2012 was 27.7 years (SD = 8.9, median = 25) with a mean of 3.9 specimens per person (SD = 3.9, median = 2) (Table 1). The mean length of time between consecutive serum specimens from the same person was 1.1 years (median = 1). The range of time between an individual’s first and last specimens went from as little as 1 day to as many as 27 years; and the mean length of time was 6.4 years (SD = 5.6).

**Table 1:** Summary statistics for individuals’ ages at the time of serum collection and the timing of collections for specimens in the DoD Serum Repository.

	Mean	Standard Deviation	Median
Age at date of collection, years^a^	27.7	8.9	25
Specimens per person	3.9	3.9	2
Years between specimens^**b**^	1.1	0.8	1
Years between first and last specimens	6.4	5.6	4

a. The maximum legal age for service members is between 17 and 62 years with few exceptions, so only those who were 17–70 years of age were included. Outliers were dropped.

b. A total of 4,642,870 individuals provided only 1 specimen.

**Fig 1:**
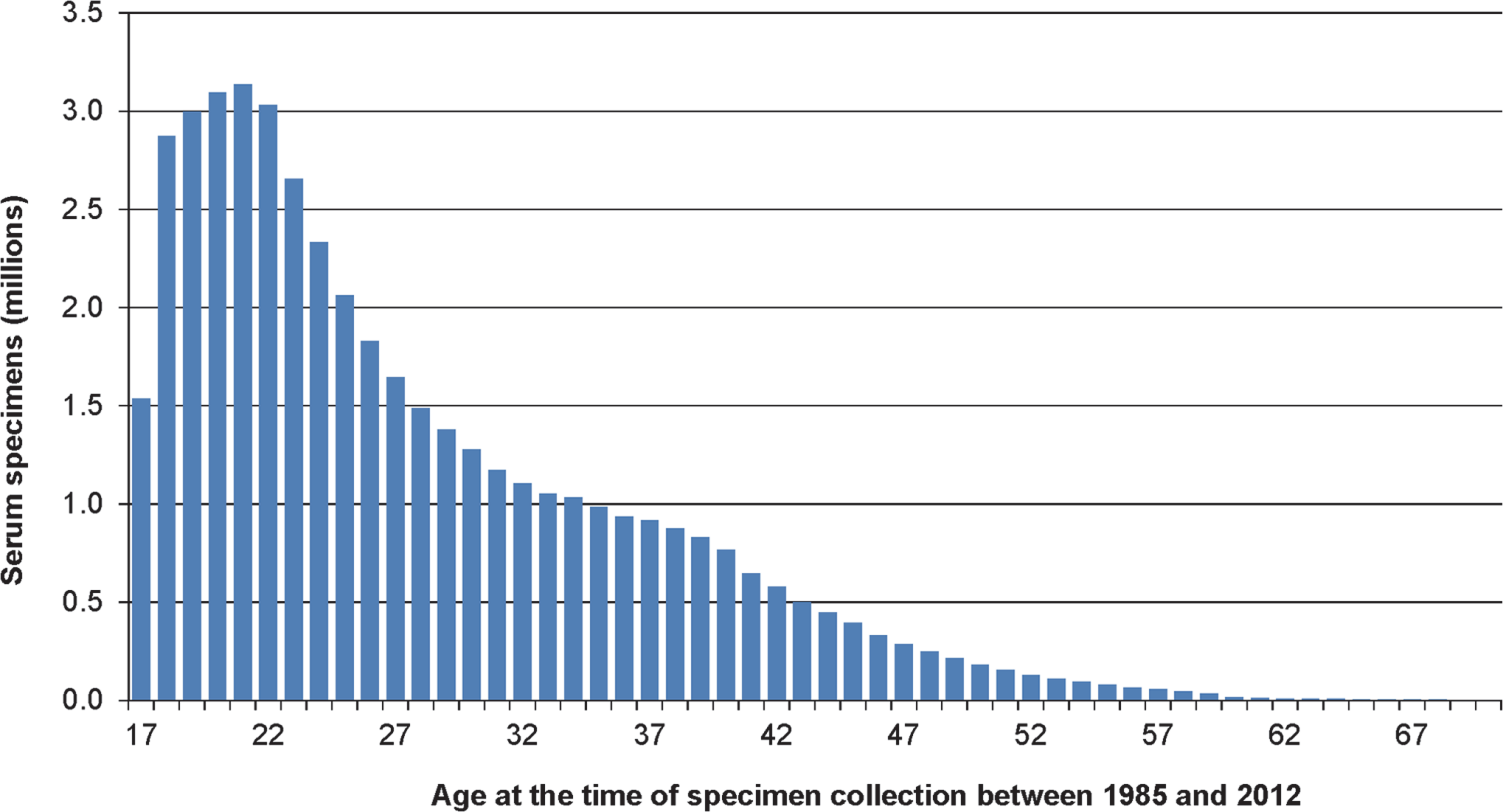
Distribution of ages^a^ at the time of collection for specimens in the DoD Serum Repository. a. Approximately 30,000 outliers younger than 17 years and older than 70 years were dropped.

The distribution of ages at the times of serum collection, depicted in Fig. 1 for those between 17 and 70 years, was heavily weighted towards the younger ages. Twenty-one-year-olds donated the greatest number of specimens (3,136,045 or 6.9%), followed by 20-, 22-, 19-, and 18-year-olds in descending order. Those under 30 years of age contributed 65.8% of all the specimens in the DoDSR between 1985 and 2012. Those over 50 years of age contributed only 1.8% of the specimens in the repository. Table 2 shows the number of people who provided multiple serum specimens between 1985 and 2012. Nearly half (47.9%) of those with serum in the DoDSR provided three or more specimens; and nearly one-fifth (19.2%) provided more than six specimens during their time in service.

**Table 2:** The numbers of individuals who provided specimens to the DoD Serum Repository and the numbers of specimens per person.

Specimens available	1	2	3	4	5	>5	Total
Number of individuals	4,642,870	1,819,626	1,156,274	959,814	808,115	3,036,255	12,422,954
%	37.4%	14.6%	9.3%	7.7%	6.5%	24.4%	100%

### Requests for Serum from the DoDSR

Using data from the DoDSR inventory management database, we were able to examine the general nature of requests for serum that occurred between 2001 and 2012. There were 325 unique requests for serum that were granted by either AMSA or AFHSC, 113 (34.8%) of which were research studies and the remaining 212 (65.2%) were for an operational (non-research) requirement. While the majority of unique requests for serum were for operational (public health or clinical) purposes, the vast majority of specimens retrieved between 2001 and 2012 (>99%, data not shown) were used for research studies.

### Publication of Studies using Specimens from the DoDSR

**Fig 2:**
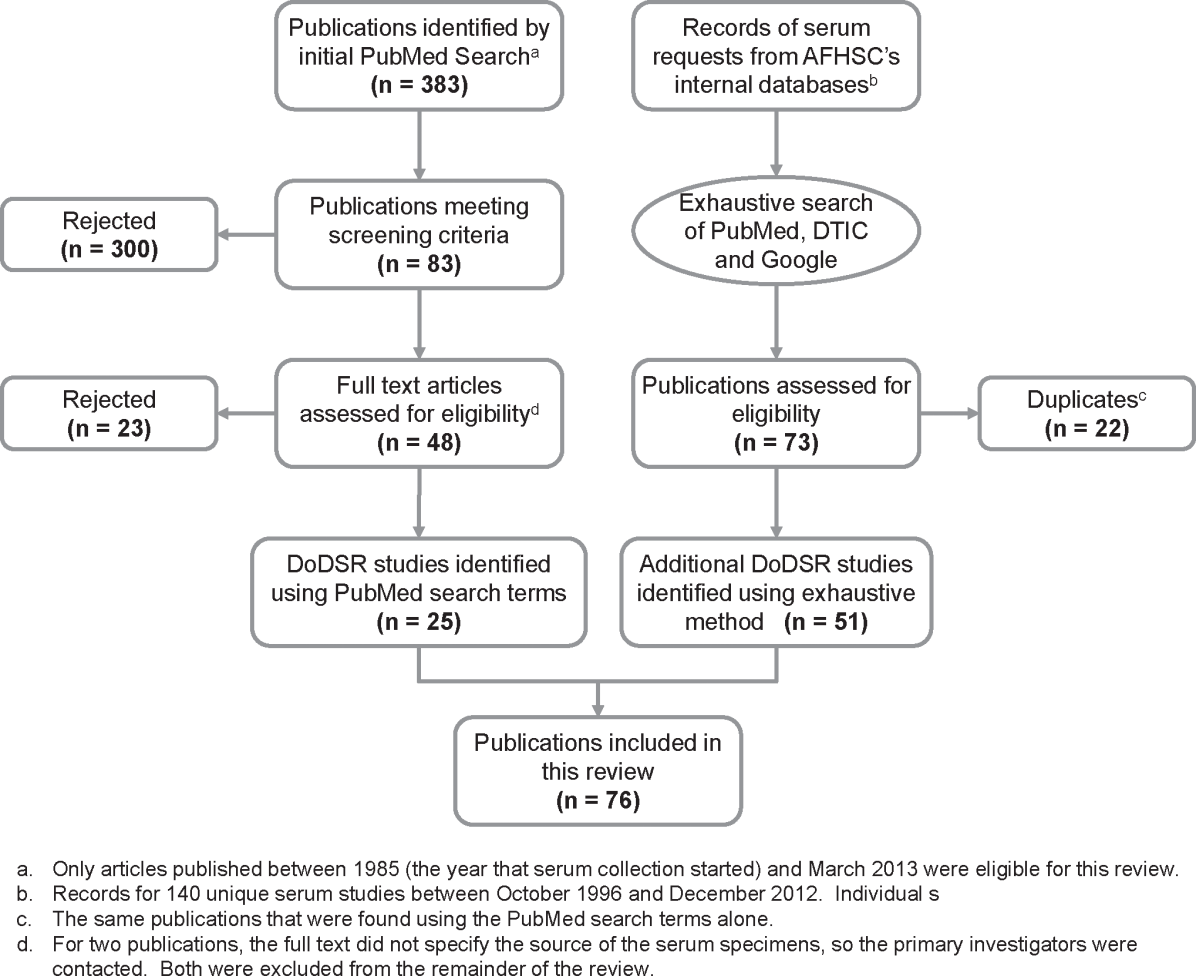
Preferred Reporting Items for Systematic Reviews and Meta-Analyses (PRISMA) flow chart for the results of the literature search using both the algorithmic and intense methods.

Searching *PubMed*, DTIC, and Google using both an algorithmic method as well as an exhaustive, highly-detailed, intense method that took advantage of the records maintained by AFHSC since 1996, we identified a total of 76 publications that used specimens from the DoDSR. Fig. 2 uses the recommend format for Preferred Reporting Items for Systematic Reviews and Meta-Analyses (PRISMA) (http://www.prisma-statement.org/) to describe the results from the two search methods that we used. None of the research studies supported in 2012 had been published as of March 2013 (data no shown). There was a substantial lag (median = 4 years) between when serum samples were retrieved (according to AFHSC records) and when an article was published (data not shown). We discovered that the results of five non-research, public health investigations were also published.[23,24,25,26,27] Although we did not specifically set out to identify non-research publications, those were included in this review because they represent important evidence of the utility of the DoDSR. A complete list of publications is available in S1 Document.

In evaluating the content of the published works, we abstracted information about the analytes, health outcomes, and risk factors addressed by the investigators. Other information about methodology and numerical values for findings (that might be used for statistical meta-analysis), were not included. We grouped all the chemokines, cytokines and hormone-like molecules as “regulatory substances.” We similarly grouped all of the “protein biomarkers” (as such) and the pathogen-specific antibodies that had unique epitopes (such as the variety of anti-Epstein-Barr virus (EBV)-induced immunoglobulins) in order to simplify the summary. Therefore, there were 59 different analytes within the 76 DoDSR publications that we identified (Table 3). A brief review of a small selection of those publications follows.

**Table 3:** Analytes^a^ from published studies that used specimens from the DoD Serum Repository and the number of articles in which each appears.

Number of articles	Analytes investigated
6	Epstein-Barr virus, 25-hydroxyvitamin D
5	Herpes simplex virus 2, human immunodeficiency virus, systemic lupus erythematosus autoantibody
4	Regulatory molecules (cytokines/chemokines/hormones)
3	Adenovirus, rheumatoid arthritis autoantibody, *Chlamydia pneumoniae*, *Coxiella burnetii*, herpes simplex virus 1, organochlorines, Prostate-specific antigen
2	Multiple sclerosis autoantibody, cytomegalovirus, hepatitis B virus, hepatitis C virus, human DNA, human herpes virus 6, influenza A virus, influenza B virus, human papillomavirus, measles virus, rubella virus, tick-borne encephalitis virus, varicella-zoster virus, “protein biomarkers”
1	*Anaplasma phagocytophilum*, mumps virus, anthrax vaccine, *Mycobacterium fermentans*, basement membrane glomerular nephropathy autoantibody, *Mycobacterium pneumonia*, thyroid autoantibody, myeloma immunoglobulin, *Bordatella pertussis*, omega-3 fatty acid (docosahexaenoic acid), *Borrelia burgdorferii*, parainfluenza virus, bovine casein (milk protein), *Plasmodium falciparum*, *Chlamydia trachomatis*, respiratory syncytial virus, dioxin, *Rickettsia rickettsiae*, diphtheria vaccine, Rift Vally fever virus, hantavirus, sandfly fever virus (Naples and Sicilian types), hepatitis A virus, Sindbis virus, Hepatitis E virus, tetanus toxoid vaccine, human T-lymphotropic virus (I and II), tick-borne encephalitis virus vaccine, influenza vaccine, *Toxoplasma gondii*, Japanese encephalitis virus vaccine, West Nile virus

a. Unless otherwise specified, mention of a microbe or vaccine refers to the human antibody to that microbe or induced by the vaccine. Antibodies to the same microbe but with different epitopes are grouped.

EBV antibodies and vitamin D were the most commonly studied analytes with six publications each. Investigators found a weak association (though not necessarily causal) between past exposure to EBV and the development of multiple sclerosis (MS),[28,29,30,31] but there were no significant associations for development of schizophrenia[32,33] or non-Hodgkin lymphoma.[34] For vitamin D, there were weak statistical associations with lower risk of breast cancer among young women,[35] lower risk of MS[36] and lower risk of stress fractures in young women (especially in the highest range of serum concentrations),[37] but the authors cautiously noted that use of vitamin D for prevention of those conditions required further study. There were no associations found between serum vitamin D concentrations in frozen serum and insulin-dependent diabetes,[38] HIV-related morbidity and mortality[39] or suicide.[40]

The DoDSR has been used to study exposures to 29 viruses, 9 bacteria, and 1 protozoan (*Plasmodium falciparum)*. The earliest retrospective serum study confirmed that the HIV testing methods used in the military between 1987 and 1988 were quite sensitive, indicating that no changes to screening policies were needed.[13] Among other findings related to infectious disease risks in the US military, DoDSR studies revealed important information about the prevalence of adenovirus infections among military recruits[17,26] and resulted in a new estimate of herpes simplex virus 1 and 2 infections in young adults.[41] *Coxiella burnetii* (the cause of Q fever) was the subject of three studies, but the investigators found no significant association between the occurrence of febrile illnesses during deployments to Iraq and development of *C*. *burnetii* antibodies afterwards.[23,42,43]

DoDSR specimens were used to establish the “baseline” rates of hepatitis A, [44] B (HBV), [45] C (HCV), [45] and E [46] virus infections among service members. Another study established that there was a relatively low risk of HIV, HCV, or HBV transmission from field blood donors during deployments to Iraq and Afghanistan. [24] DoDSR specimens were also used to determine that exposures to certain infectious agents—Mycoplasma *fermentans*, [47] tick-borne encephalitis virus, [18, 48] numerous arborviruses, [49] and several rickettsiae [48]—were relatively uncommon during military service (including deployments). One publication reported on attempts to identify serum analytes that could be used to confirm *P*. *falciparum* exposure among those who had deployed to northern Africa. [50]

Numerous other studies and findings have had health implications beyond the military population. Two studies helped to establish race-specific screening values for prostate-specific antigen (PSA) in young men.[51,52] Among findings most generalizable to the US population, several studies expanded the search for serum biomarkers in young adults that could be used to predict the subsequent onset of chronic illnesses such as systemic lupus erythematosus,[53,54,55,56,57,58] rheumatoid arthritis,[59,60,61] multiple sclerosis,[30,62] schizophrenia,[32,33,63,64,65] and non-Hodgkin lymphoma.[66]

Only a few published studies addressed non-protein analytes. Among those, Taylor and colleagues found insignificant amounts of dioxin in the stored serum of a small number of individuals (*n* = 5) who had been exposed to burn bits in Iraq.[27] McGlynn and Chia (separately) and their respective teams found no association between serum organochlorines and future development of germ cell testicular cancers.[67,68,69] Another non-protein study found a weak association between the lowest concentrations of omega-3-fatty acid and suicidal behaviors.[70] Similar to the vitamin D studies, the investigators noted that more study would be needed to test the hypothesis that fatty acid supplementation could reduce suicides.

Only two published studies attempted to detect human genetic material in frozen serum. One study involved those who developed PTSD and provided weak evidence that deoxyribonucleic acid methylation might be a sign of, or contributory factor to, mental resiliency against combat stress. [71] The other study provided support for the hypothesis that those with a gene-variant of the methylenetetrahydrofolate reductase (MTHFR) enzyme (and subsequently at risk for lower serum folate) were at increased risk of seizures following head injury. [72]

Table 4 compares the types of analytes identified in DoDSR-related publications (from Table 3) with the types of health outcomes that were the subject of study. Several publications included more than one type of analyte; and some studies examined the association between an analyte and more than one type of outcome. Infectious diseases were the most common type of health outcome studied (36.8%)—mostly (93.3%) through the detection of human, anti-pathogen immunoglobulins. Autoimmune disorders were the next most common type of health outcome studied (26.3%), followed by neoplasms (14.5%), mental health disorders (10.5%), and vaccine-induced immunity (5.3%).

**Table 4:** Number of publications stratified by types of health outcomes studied using specimens from the DoD Serum Repository and the analytes investigated in those studies.

Health Outcome	Antibodies	Regulatory molecules and Biomarkers	Chemical compounds	Human DNA	Nutrient	Total
Infectious diseases	28	1			1	30^a^
Neoplasms	3	4	3		1	11
Chronic metabolic	1				1	2
Autoimmune disorders	17	2			1	20^b^
Non-specific “war syndrome”	1		1			2
Vaccine-induced immunity	4					4^a^
Physical injury					1	1
Neurological				1		1
Mental illness	3	2		1	2	8
Total	57^a^	8^b^	4	2	7	76

a. Two publications [18, 74] addressed both the incidence of acute infection (as indicated by the presence of pathogen-induced antibody) and vaccine-induced antibody. In the table, this affects the row and columns totals but not the grand total.

b. One publication [59] assayed for an autoantibody as well as the presence of disease progression markers (various cytokines and chemokines). In the table, this affects the row and columns totals, but not the grand total.

## Discussion

Despite using less than one-half of one percent of the available specimens in the DoDSR, military investigators have contributed important information on a wide variety of pathogens, exposures, and disease processes. Among the relevant findings were the baseline prevalence or incidence of a number of infectious and non-infectious conditions that can impact military forces. Examples include Q fever, viral hepatitides, respiratory infections (including adenovirus and influenza), a variety of arboviruses specific to Central Asia, human papillomavirus and rickettsioses. The DoDSR has been used to study risk factors for suicide, PTSD, early-onset diabetes, stress fractures (which are relatively common in military trainees [73]) and a number of cancers that can have early age of onset. There have been key studies informing military policies for vaccination against adenovirus, anthrax, influenza, hepatitis A and B, Japanese encephalitis virus, tick-borne encephalitis virus, measles, mumps, and rubella; and for policies related to the DoD’s donor blood and force health programs.

A number of studies took innovative approaches that highlight the unique value of the DoDSR. Faix and colleagues [74] used banked serum to evaluate immunological responses to the 2011 strain of influenza A(H1N1) virus and the 2011 vaccine among those who had been previously exposed to pandemic influenza A(H1N1) in 2009. They calculated a relatively low vaccine effectiveness, which suggested that genetic drift of the influenza virus over the course of two years was sufficient to lead to reduced vaccine efficacy, a conclusion that could have significant implications for influenza vaccine policy. Schwarz and colleagues, [64] in a very large, retrospective case-control study of service members with schizophrenia and bipolar diagnoses identified a number of serum proteins that may aid in the diagnosis of those complex health outcomes. Several studies using specimens from the DoDSR pointed to the possibility that nutritional supplementation may help prevent suicides and stress fractures. Those and other research projects used study designs that would have been very difficult or very expensive without the DoDSR as a resource.

Genetic studies using the DoDSR are likely limited because serum contains very little genetic material and specimens are stored only at -30°C. The DoDSR currently encompasses nearly 450,000 cubic feet (12,743 cubic meters) of frozen storage—roughly equivalent to 11 high school basketball courts with 12-foot ceilings (M.V. Rubertone, AFHSC, personal communication)—so it seems unlikely that the entire facility can be affordably maintained colder than -30°C. However, the two published studies we identified that analyzed genetic material [71, 72] suggest that it may be possible to detect small gene segments sufficient for some types of studies.

Given that nearly two-thirds of all requests for serum since 2001 were for either a clinical study or a public health investigation, it seems clear that the DoDSR is fulfilling its primary mission. However, for unpredictable or rare health outcomes—PTSD, autoimmune disorders, cancers, vaccine adverse events, psychiatric disorders, sequelae of traumatic brain injuries, long-term outcomes from certain infectious diseases (such as post-Q fever cardiomyopathy) or low-level toxin exposures—the DoDSR could be an unparalleled resource for medical research. Our review of the uses of the DoDSR confirms its scientific value, yet also suggests that limiting the use of the DoDSR to military investigators also limits the beneficial uses.

Whether greater use of the DoDSR is allowable is a public policy decision, however, not strictly a scientific one. Confidentiality and the future availability of serum for operational purposes, including individual healthcare, is paramount, but there could be ways to increase use of stored serum without jeopardizing the healthcare or privacy of service members. At the very least, US military policies would need to be modified to allow non-military investigators (using non-military HSRBs) to use the DoDSR.

With access to frozen serum specimens, the US Department of Veterans Affairs and other institutions (both public and private) could potentially study a wide range of conditions related to past military service as well as outcomes that are the result of lifestyle factors, occupational and non-occupational exposures, or the natural history of human aging. It may be possible that conditions with onsets later in life (such as essential hypertension, cancers or Alzheimer’s disease) could be better understood through the study of biological precursors in “younger” serum. The policies of the researchers’ institutions (external to the DoD) would need to support the exchange of personal identifiers in order to explore the availability of specimens and to create valid study designs. Researchers who make the effort to enroll former service members could ultimately be given access to subjects’ specimens if they have a non-military, HSRB-approved protocol.

There are 12.42 million people represented in the DoDSR, so it seems likely that there are sufficient numbers of subjects (and serum samples) for many, many more studies. However, there may be other legal or regulatory obstacles of which we are not aware at this time. To guide policy decisions, another area of study could be to determine the value that individual service members and policy makers place on the repository and what additional uses (and users) would be acceptable.

There is a constant inflow of specimens into the repository, and AFHSC is limited in its ability to support very large numbers of studies simultaneously. Specimens are retrieved from the laboratories (after HIV testing) using a dedicated refrigerator truck, catalogued prior to placement; taken from storage manually; thawed and aliquoted in a small lab using a robotic system, then placed back into storage by the DoDSR staff. More human resources and specialized equipment would be needed to increase the speed of cataloguing, retrieval and processing if more studies are to be supported.

There are some limitations to the methods we used. Without systematically contacting the investigators, it is impossible to know what they found or why their results were not published. Perhaps their targeted analytes could not be detected, which is information that could affect our understanding of the utility of the DoDSR. An informal review of the research studies that had not been published as of April 2013 revealed no pattern of health conditions or analytes different from those that had been published (unpublished data). Additionally, we did not include the several meeting abstracts that we discovered; and we made no attempt to find DoDSR-related findings in book format. It is also possible that we missed some studies because we did not search through paper and email records for years prior to 1996. However, given that official guidance to conduct medical research using the DoDSR was not published until 1997, we believe that doing so would have resulted in a small number of additional publications at worst. Overall, we believe that these limitations had very little impact on our conclusions.

We were somewhat surprised that using PubMed was not a very effective means for identifying DoDSR-related publications. The advantage of complementing the algorithmic approach with the intense approach was that we are very confident that we found almost every publication in existence; the disadvantage is that work to discover publications necessarily had to stop in April 2013 in order to dedicate time to analysis and publication. To attempt to improve the ability to identify publications and other scientific results from use of the DoDSR, in 2013, AFHSC update the research study support letters to require specific statements in manuscripts about the source of data and specimens; and to provide presentations and publications to the AFHSC for review prior to release. However, it will still be difficult for AFHSC to enforce these requirements beyond banning future support of investigators who do not abide by them. Therefore, we appeal to former and future researchers who receive specimens from the DoDSR to acknowledge the benefit that they receive from the investment by the DoD; as well as from the members of the Armed Forces who provided specimens.

## Conclusions

The DoD Serum Repository is an invaluable asset for the military health system and the population it serves. Whether during the active duty period or afterwards, Service members’ frozen serum specimens are available for medical testing if needed, but the repository is also an important resource that can help Service members and healthcare providers to understand the health consequences of military service. Studies using reposed serum samples can help to illuminate the pathogenesis of diseases and conditions that affect the military and former-military populations, whether as the consequence of occupational exposures or the natural history of disease. There have been a number of important findings that would not have been possible without a facility like the DoDSR. Despite the occupational health insights and scientific progress already gained through use of the DoDSR, it has been underutilized. Strictly limiting use of the repository to military investigators protects the DoDSR’s fundamental purpose, but also limits the number and type of investigations that can be accomplished. Controlled access to the DoDSR for civilian scientists who are working on militarily relevant problems would be ideal, but new administrative controls, enhanced specimen processing and updated regulatory guidelines would be needed. More study is needed to understand the limitations of using long-frozen serum for health research, and more can be done to determine the social value of the DoDSR. The DoD Serum Repository will continue to grow as HIV screening and deployment-related serum collections continue, so decisions about the repository’s future are inevitable.

## Supporting Information

S1 Document References for articles published between 1988 and February 2013 describing studies that used thawed specimens from the DoD Serum Repository (DoDSR) in roughly chronological order. (DOCX)

S1 PRISMA Checklist Preferred Reporting Items for Systematic Reviews and Meta-Analyses (PRISMA) (http://www.prisma-statement.org/) checklist. (DOC)
