# Supplementary material for: Description and Utilization of the United States Department of Defense Serum Repository: A Review of Published Studies, 1985-2012
Source: PLoS One. 2015 Feb 27;10(2):e0114857. doi: 10.1371/journal.pone.0114857 (PMC4344338; doi:10.1371/journal.pone.0114857)
Supplement: S1 Document — (DOCX) [file pone.0114857.s001.docx]

**Document S1**

1. Burke DS, Brundage JF, Redfield RR, Damato JJ, Schable CA, et al. (1988) Measurement of the false positive rate in a screening program for human immunodeficiency virus infections. New England Journal of Medicine 319: 961-964.

2. Roberts CR, Fipps DR, Brundage JF, Wright SE, Goldenbaum M, et al. (1992) Prevalence of human T-lymphotropic virus in civilian applicants for the United States Armed Forces. American Journal of Public Health 82: 70-73.

3. Kelley PW (1994) Susceptibility to measles and reubella among U.S. Army recruits : a seroepidemiologic analysis of risk factors, temporal trends, and disease control policy options [Dissertation/Thesis]. Baltimore, MD: Johns Hopkins University. xv, 199 p.

4. Gambel JM, DeFraites R, Hoke C, Jr., Brown A, Sanchez J, et al. (1995) Japanese encephalitis vaccine: persistence of antibody up to 3 years after a three-dose primary series. Journal of Infectious Diseases 171: 1074.

5. Ludwig SL, Brundage JF, Kelley PW, Nang R, Towle C, et al. (1998) Prevalence of antibodies to adenovirus serotypes 4 and 7 among unimmunized US Army trainees: results of a retrospective nationwide seroprevalence survey. Journal of Infectious Diseases 178: 1776-1778.

6. Craig SC, Pittman PR, Lewis TE, Rossi CA, Henchal EA, et al. (1999) An accelerated schedule for tick-borne encephalitis vaccine: the American Military experience in Bosnia. American Journal of Tropical Medicine and Hygiene 61: 874-878.

7. Lo SC, Levin L, Ribas J, Chung R, Wang RY, et al. (2000) Lack of serological evidence for Mycoplasma fermentans infection in army Gulf War veterans: a large scale case-control study. Epidemiology and Infection 125: 609-616.

8. Preston DM, Levin LI, Jacobson DJ, Jacobsen SJ, Rubertone M, et al. (2000) Prostate-specific antigen levels in young white and black men 20 to 45 years old. Urology 56: 812-816.

9. Arbuckle MR, James JA, Kohlhase KF, Rubertone MV, Dennis GJ, et al. (2001) Development of anti-dsDNA autoantibodies prior to clinical diagnosis of systemic lupus erythematosus. Scandinavian Journal of Immunology 54: 211-219.

10. Barker TL, Richards AL, Laksono E, Sanchez JL, Feighner BH, et al. (2001) Serosurvey of Borrelia burgdorferi infection among U.S. military personnel: a low risk of infection. American Journal of Tropical Medicine and Hygiene 65: 804-809.

11. Hyams KC, Riddle J, Rubertone M, Trump D, Alter MJ, et al. (2001) Prevalence and incidence of hepatitis C virus infection in the US military: a seroepidemiologic survey of 21,000 troops. American Journal of Epidemiology 153: 764-770.

12. Sanchez JL, Jr., Craig SC, Kohlhase K, Polyak C, Ludwig SL, et al. (2001) Health assessment of U.S. military personnel deployed to Bosnia-Herzegovina for operation joint endeavor. Military Medicine 166: 470-474.

13. Arbuckle MR, James JA, Dennis GJ, Rubertone MV, McClain MT, et al. (2003) Rapid clinical progression to diagnosis among African-American men with systemic lupus erythematosus. Lupus 12: 99-106.

14. Arbuckle MR, McClain MT, Rubertone MV, Scofield RH, Dennis GJ, et al. (2003) Development of autoantibodies before the clinical onset of systemic lupus erythematosus. New England Journal of Medicine 349: 1526-1533.

15. Levin LI, Munger KL, Rubertone MV, Peck CA, Lennette ET, et al. (2003) Multiple sclerosis and Epstein-Barr virus (RETRACTED). Journal of the American Medical Association 289: 1533-1536.

16. McClain MT, Arbuckle MR, Heinlen LD, Dennis GJ, Roebuck J, et al. (2004) The prevalence, onset, and clinical significance of antiphospholipid antibodies prior to diagnosis of systemic lupus erythematosus. Arthritis and Rheumatism 50: 1226-1232.

17. Munger KL, DeLorenze GN, Levin LI, Rubertone MV, Vogelman JH, et al. (2004) A prospective study of Chlamydia pneumoniae infection and risk of MS in two US cohorts. Neurology 62: 1799-1803.

18. Anderson AD, Smoak B, Shuping E, Ockenhouse C, Petruccelli B (2005) Q fever and the US military. Emerging Infectious Diseases 11: 1320-1322.

19. Arcari CM, Gaydos CA, Nieto FJ, Krauss M, Nelson KE (2005) Association between Chlamydia pneumoniae and acute myocardial infarction in young men in the United States military: the importance of timing of exposure measurement. Clinical Infectious Diseases 40: 1123-1130.

20. Levin LI, Munger KL, Rubertone MV, Peck CA, Lennette ET, et al. (2005) Temporal relationship between elevation of epstein-barr virus antibody titers and initial onset of neurological symptoms in multiple sclerosis. Journal of the American Medical Association 293: 2496-2500.^[[1]](#footnote-1)^

21. Scott PT, Niebuhr DW, McGready JB, Gaydos JC (2005) Hepatitis B immunity in United States military recruits. Journal of Infectious Diseases 191: 1835-1841.

22. Munger KL, Levin LI, Hollis BW, Howard NS, Ascherio A (2006) Serum 25-hydroxyvitamin D levels and risk of multiple sclerosis. Journal of the American Medical Association 296: 2832-2838.

23. Nevin RL, Niebuhr DW (2007) Rising hepatitis A immunity in U.S. military recruits. Military Medicine 172: 787-793.

24. Chia VM, Quraishi SM, Graubard BI, Rubertone MV, Erickson RL, et al. (2008) Insulin-like growth factor 1, insulin-like growth factor-binding protein 3, and testicular germ-cell tumor risk. Am J Epidemiol 167: 1438-1445.

25. Eick AA, Hu Z, Wang Z, Nevin RL (2008) Incidence of mumps and immunity to measles, mumps and rubella among US military recruits, 2000-2004. Vaccine 26: 494-501.

26. Faix DJ, Harrison DJ, Riddle MS, Vaughn AF, Yingst SL, et al. (2008) Outbreak of Q fever among US military in western Iraq, June-July 2005. Clinical Infectious Diseases 46: e65-68.

27. Graf PC, Chretien JP, Ung L, Gaydos JC, Richards AL (2008) Prevalence of seropositivity to spotted fever group rickettsiae and Anaplasma phagocytophilum in a large, demographically diverse US sample. Clinical Infectious Diseases 46: 70-77.

28. Majka DS, Deane KD, Parrish LA, Lazar AA, Baron AE, et al. (2008) Duration of preclinical rheumatoid arthritis-related autoantibody positivity increases in subjects with older age at time of disease diagnosis. Annals of the Rheumatic Diseases 67: 801-807.

29. McGlynn KA, Quraishi SM, Graubard BI, Weber JP, Rubertone MV, et al. (2008) Persistent organochlorine pesticides and risk of testicular germ cell tumors. Journal of the National Cancer Institute 100: 663-671.

30. Niebuhr DW, Millikan AM, Cowan DN, Yolken R, Li Y, et al. (2008) Selected infectious agents and risk of schizophrenia among U.S. military personnel. Am Journal of Psychiatry 165: 99-106.

31. Niebuhr DW, Millikan AM, Yolken R, Li Y, Weber NS (2008) Results from a hypothesis generating case-control study: herpes family viruses and schizophrenia among military personnel. Schizophrenia Bulletin 34: 1182-1188.

32. Riddle MS, Althoff JM, Earhart K, Monteville MR, Yingst SL, et al. (2008) Serological evidence of arboviral infection and self-reported febrile illness among U.S. troops deployed to Al Asad, Iraq. Epidemiology and Infection 136: 665-669.

33. Singer DE, Schneerson R, Bautista CT, Rubertone MV, Robbins JB, et al. (2008) Serum IgG antibody response to the protective antigen (PA) of Bacillus anthracis induced by anthrax vaccine adsorbed (AVA) among U.S. military personnel. Vaccine 26: 869-873.

34. Taylor G, Rush V, Deck A, Vietas JA (2008) Screening Health Risk Assessment Burn Pit Exposures, Balad Air Base, Iraq and Addendum Report. Brooks City-Base, TX: Air Force Institute for Operational Health, Risk Analysis Directorate, Health and Safety Division. 134 p.

35. Wang H, Munger KL, Reindl M, O'Reilly EJ, Levin LI, et al. (2008) Myelin oligodendrocyte glycoprotein antibodies and multiple sclerosis in healthy young adults. Neurology 71: 1142-1146.

36. Bautista CT, Singer DE, O"Connell RJ, Crum-Cianflone N, Agan BK, et al. (2009) Herpes simplex virus type 2 and HIV infection among US military personnel: implications for health prevention programmes. International Journal of STD & AIDS 20: 634-637.

37. Dennis LK, Coughlin JA, McKinnon BC, Wells TS, Gaydos CA, et al. (2009) Sexually transmitted infections and prostate cancer among men in the U.S. military. Cancer Epidemiology, Biomarkers and Prevention 18: 2665-2671.

38. Massa J, Munger KL, O'Reilly EJ, Levin LI, Ascherio A (2009) Serum titers of IgG antibodies against tetanus and diphtheria toxoids and risk of multiple sclerosis. Journal of Neuroimmunology 208: 141-142.

39. McGlynn KA, Quraishi SM, Graubard BI, Weber JP, Rubertone MV, et al. (2009) Polychlorinated biphenyls and risk of testicular germ cell tumors. Cancer Research 69: 1901-1909.

40. Tate JE, Bunning ML, Lott L, Lu X, Su J, et al. (2009) Outbreak of severe respiratory disease associated with emergent human adenovirus serotype 14 at a US air force training facility in 2007. Journal of Infectious Diseases 199: 1419-1426.

41. Weiss BM, Abadie J, Verma P, Howard RS, Kuehl WM (2009) A monoclonal gammopathy precedes multiple myeloma in most patients. Blood 113: 5418-5422.

42. Anderson AD, Baker TR, Littrell AC, Mott RL, Niebuhr DW, et al. (2010) Seroepidemiologic survey for Coxiella burnetii among hospitalized US troops deployed to Iraq. Zoonoses and Public Health 58: 276-283.

43. Chia VM, Li Y, Quraishi SM, Graubard BI, Figueroa JD, et al. (2010) Effect modification of endocrine disruptors and testicular germ cell tumour risk by hormone-metabolizing genes. International Journal of Andrology 33: 588-596.

44. Deane KD, O'Donnell CI, Hueber W, Majka DS, Lazar AA, et al. (2010) The number of elevated cytokines and chemokines in preclinical seropositive rheumatoid arthritis predicts time to diagnosis in an age-dependent manner. Arthritis and Rheumatism 62: 3161-3172.

45. Eick A, Ticehurst J, Tobler S, Nevin R, Lindler L, et al. (2010) Hepatitis E seroprevalence and seroconversion among US military service members deployed to Afghanistan. Journal of Infectious Diseases 202: 1302-1308.

46. Heinlen LD, McClain MT, Ritterhouse LL, Bruner BF, Edgerton CC, et al. (2010) 60 kD Ro and nRNP A frequently initiate human lupus autoimmunity. PLoS One 5: e9599.

47. Heinlen LD, Ritterhouse LL, McClain MT, Keith MP, Neas BR, et al. (2010) Ribosomal P autoantibodies are present before SLE onset and are directed against non-C-terminal peptides. Journal of Molecular Medicine 88: 719-727.

48. Kolfenbach JR, Deane KD, Derber LA, O'Donnell CI, Gilliland WR, et al. (2010) Autoimmunity to peptidyl arginine deiminase type 4 precedes clinical onset of rheumatoid arthritis. Arthritis and Rheumatism 62: 2633-2639.

49. Levin LI, Munger KL, O'Reilly EJ, Falk KI, Ascherio A (2010) Primary infection with the Epstein-Barr virus and risk of multiple sclerosis. Annals of Neurology 67: 824-830.

50. Schwarz E, Izmailov R, Spain M, Barnes A, Mapes JP, et al. (2010) Validation of a blood-based laboratory test to aid in the confirmation of a diagnosis of schizophrenia. Biomarker Insights 5: 39-47.

51. Singer DE, Bautista CT, O'Connell RJ, Sanders-Buell E, Agan BK, et al. (2010) HIV infection among U.S. Army and Air Force military personnel: sociodemographic and genotyping analysis. AIDS Research and Human Retroviruses 26: 889-894.

52. Burgi AA, Gorham ED, Garland CF, Mohr SB, Garland FC, et al. (2011) High serum 25-hydroxyvitamin D is associated with a low incidence of stress fractures. Journal of Bone and Mineral Research 26: 2371-2377.

53. Campo JJ, Whitman TJ, Freilich D, Burgess TH, Martin GJ, et al. (2011) Toward a surrogate marker of malaria exposure: modeling longitudinal antibody measurements under outbreak conditions. PLoS One 6: e21826.

54. Eick AA, Faix DJ, Tobler SK, Nevin RL, Lindler LE, et al. (2011) Serosurvey of bacterial and viral respiratory pathogens among deployed U.S. service members. American Journal of Preventive Medicine 41: 573-580.

55. Hakre S, Peel SA, O'Connell RJ, Sanders-Buell EE, Jagodzinski LL, et al. (2011) Transfusion-transmissible viral infections among US military recipients of whole blood and platelets during Operation Enduring Freedom and Operation Iraqi Freedom. Transfusion 51: 473-485.

56. Hutfless S, Matos P, Talor MV, Caturegli P, Rose NR (2011) Significance of prediagnostic thyroid antibodies in women with autoimmune thyroid disease. Journal of Clinical Endocrinology and Metabolism 96: E1466-1471.

57. Lewis MD, Hibbeln JR, Johnson JE, Lin YH, Hyun DY, et al. (2011) Suicide deaths of active-duty US military and omega-3 fatty-acid status: a case-control comparison. Journal of Clinical Psychiatry 72: 1585-1590.

58. Munger KL, Levin LI, O'Reilly EJ, Falk KI, Ascherio A (2011) Anti-Epstein-Barr virus antibodies as serological markers of multiple sclerosis: a prospective study among United States military personnel. Multiple Sclerosis 17: 1185-1193.

59. Niebuhr DW, Li Y, Cowan DN, Weber NS, Fisher JA, et al. (2011) Association between bovine casein antibody and new onset schizophrenia among US military personnel. Schizophrenia Research 128: 51-55.

60. Olson SW, Arbogast CB, Baker TP, Owshalimpur D, Oliver DK, et al. (2011) Asymptomatic autoantibodies associate with future anti-glomerular basement membrane disease. Journal of American Society of Nephrology 22: 1946-1952.

61. Scher AI, Wu H, Tsao JW, Blom HJ, Feit P, et al. (2011) MTHFR C677T genotype as a risk factor for epilepsy including post-traumatic epilepsy in a representative military cohort. Journal of Neurotrauma 28: 1739-1745.

62. Sutcliffe S, Nevin RL, Pakpahan R, Elliott DJ, Cole SR, et al. (2011) Prostate involvement during sexually transmitted infections as measured by prostate-specific antigen concentration. British Journal of Cancer 105: 602-605.

63. Vendrame E, Martinez-Maza O (2011) Assessment of pre-diagnosis biomarkers of immune activation and inflammation: insights on the etiology of lymphoma. Journal of Proteome Research 10: 113-119.

64. Brett-Major DM, Hakre S, Naito NA, Armstrong A, Bower EA, et al. (2012) Epidemiology of contemporary seroincident HIV infection in the Navy and Marine corps. Military Medicine 177: 1328-1334.

65. Faix DJ, Hawksworth AW, Myers CA, Hansen CJ, Ortiguerra RG, et al. (2012) Decreased serologic response in vaccinated military recruits during 2011 correspond to genetic drift in concurrent circulating pandemic A/H1N1 viruses. PLoS One 7: e34581.

66. Gorham ED, Garland CF, Burgi AA, Mohr SB, Zeng K, et al. (2012) Lower prediagnostic serum 25-hydroxyvitamin D concentration is associated with higher risk of insulin-requiring diabetes: a nested case-control study. Diabetologia 55: 3224-3227.

67. Levin LI, Chang ET, Ambinder RF, Lennette ET, Rubertone MV, et al. (2012) Atypical prediagnosis Epstein-Barr virus serology restricted to EBV-positive Hodgkin lymphoma. Blood 120: 3750-3755.

68. Page WF, Chubb M, Feng X, Fan LY, Li Y, et al. (2012) National estimates of seroincidence and seroprevalence for herpes simplex virus type 1 and type 2 among US military adults aged 18 to 29 years. Sexually Transmitted Diseases 39: 241-250.

69. Rusiecki JA, Chen L, Srikantan V, Zhang L, Yan L, et al. (2012) DNA methylation in repetitive elements and post-traumatic stress disorder: a case-control study of US military service members. Epigenomics 4: 29-40.

70. Schwarz E, Guest PC, Rahmoune H, Martins-de-Souza D, Niebuhr DW, et al. (2012) Identification of a blood-based biological signature in subjects with psychiatric disorders prior to clinical manifestation. World Journal of Biological Psychiatry 13: 627-632.

71. Scott PT, Hakre S, Myles O, Sanders-Buell EE, Kijak GH, et al. (2012) Short communication: Investigation of incident HIV infections among U.S. army soldiers deployed to Afghanistan and Iraq, 2001-2007. AIDS Research and Human Retroviruses 28: 1308-1312.

72. Sherwood JE, Mesner OC, Weintrob AC, Hadigan CM, Wilkins KJ, et al. (2012) Vitamin D deficiency and its association with low bone mineral density, HIV-related factors, hospitalization, and death in a predominantly black HIV-infected cohort. Clinical Infectious Diseases 55: 1727-1736.

73. Sokolove J, Bromberg R, Deane KD, Lahey LJ, Derber LA, et al. (2012) Autoantibody epitope spreading in the pre-clinical phase predicts progression to rheumatoid arthritis. PLoS One 7: e35296.

74. Sutcliffe S, Pakpahan R, Sokoll LJ, Elliott DJ, Nevin RL, et al. (2012) Prostate-specific antigen concentration in young men: new estimates and review of the literature. BJU International 110: 1627-1635.

75. Agan BK, Macalino GE, Nsouli-Maktabi H, Wang X, Gaydos JC, et al. (2013) Human papillomavirus seroprevalence among men entering military service and seroincidence after ten years of service. Medical Surveillance Monthly Report 20: 21-24.

76. Mohr SB, Gorham ED, Alcaraz JE, Kane CI, Macera CA, et al. (2013) Serum 25-hydroxyvitamin D and breast cancer in the military: a case-control study utilizing pre-diagnostic serum. Cancer Causes and Control 24: 495-504.

77. Umhau JC, George DT, Heaney RP, Lewis MD, Ursano RJ, et al. (2013) Low vitamin D status and suicide: a case-control study of active duty military service members. PLoS One 8: e51543.

1. This publication replaced the 2003 paper by the same authors that had been retracted. [↑](#footnote-ref-1)
